# Physical fitness decline and career paths: a longitudinal study of medical undergraduates

**DOI:** 10.1186/s12909-024-05493-0

**Published:** 2024-05-08

**Authors:** Haitao Tang, Jinsong Wang, Ji Bao, Lie Zhang

**Affiliations:** 1 Health Management Center, General Practice Medical Center, West China Hospital, Chengdu, China; 2 Department of Postgraduate Students, West China Hospital, Sichuan University, Chengdu, China; 3 Department of Rehabilitation Medicine, West China Hospital, Sichuan University, Chengdu, China; 4 Department of Student Affairs, West China Hospital, Sichuan University, Chengdu, China; 5 Department of Pathology, Institute of Clinical Pathology, Key Laboratory of Transplant Engineering and Immunology, West China Hospital, Sichuan University, Chengdu, China; 6 Department of Emergency Management Office, West China Hospital, Sichuan University, Chengdu, China

**Keywords:** Physical fitness decline, Medical undergraduates, Career paths, Physical exercise, Health impact

## Abstract

**Introduction:**

Exercise enhances one's health and competitiveness. A strong physical fitness status can pave the way for a promising future. This study presents the time-based trends in physical fitness indicators—including height, weight, BMI, lung capacity, dash, long-distance running, and standing long jump—among medical undergraduates during their university years. Additionally, we analyzed the impact of students' physical fitness on their career paths.

**Method:**

We conducted a retrospective database study by collecting physical fitness test data and career paths information for 634 medical students from a university in southwestern China. These students graduated in 2022. The career paths included pursuits in further studies, employment, and unemployment. To detect differences in these aspects, we used the t-test and Chi-square test.

**Results:**

Our study indicates a significant declining trend in the physical fitness of medical students during their university years. The changes observed between the first and fourth tests are as follows:

- Weight (kg): 58.52 ± 10.48 to 60.73 ± 12.07, *P* < 0.00
- BMI (kg/m^2): 20.79 ± 2.74 to 21.24 ± 3.06, *P* < 0.00
- 50-m dash (s): 8.91 ± 0.99 to 9.25 ± 1.11, *P* < 0.00
- Standing long jump (cm): 187.74 ± 30.98 to 182.59 ± 32.25, *P* < 0.00
- 800-m run for females (min): 3.84 ± 0.47 to 4.48 ± 0.85, *P* < 0.00
- 1000-m run for males (min): 3.98 ± 0.63 to 4.62 ± 0.87, *P* < 0.00
- Sit-ups for females (count): 30.39 ± 7.5 to 29.03 ± 8.82, *P* < 0.00

Upon analyzing the correlation between changes in physical fitness and career paths, students with stable or decreased BMI had better post-graduation outcomes compared to students with increased BMI.

**Conclusions:**

Medical students show a declining trend in physical fitness during their undergraduate years. A good physical health status is beneficial for achieving better career paths. Medical students should place greater emphasis on physical exercise during their time in school.

## Introduction

Physical fitness plays a pivotal role in students' psychological health, academic, and professional performance [1–5]. Physical fitness, as a cornerstone of a healthy lifestyle for college students, is increasingly recognized as a key element in fostering their academic and professional advancement. Hillman, Erickson, and Kramer (2008) have shown that students who regularly engage in physical exercise perform better in memory-related tasks [6]. In line with this, Tomporowski, Lambourne, & Okumura (2011) noted that students frequently undergoing fitness training demonstrate higher problem-solving and innovation skills within projects [7]. Consistently, Stephens et al. (2015) identified physical fitness as an effective predictor of success among medical students [8]. In recent years, studies by Hou et al. (2020) have underscored the significant impact of fitness on medical students' academic outcomes [1], and research by Macky et al. (2021) suggests potential influences on clinical examination performances [9]. Furthermore, Smith & Smith (2014) discerned a positive correlation between physical fitness and students' self-esteem, self-efficacy, and emotional well-being [10].

University students’ physical fitness attracted the emphasis and measures of whole world [11, 12]. Nations worldwide have duly recognized the significance of physical fitness among their student populations and have, in response, formulated a slew of relevant policies [13]. To elevate the physical fitness levels of students, countries commonly integrate physical education and fitness training into curricula [14–17]. A report by the World Health Organization (2018) indicates that numerous countries are encouraging university students to actively participate in sports activities [18]. According to a study by the European Commission (2019), systematic fitness training has enhanced the academic performance of European university students, showing an approximate improvement of 15% [19].

There is, however, an underexplored domain: the relationship between physical fitness and career paths. While the importance of physical fitness among college students is widely acknowledged, its influence on students' career paths remains relatively underexplored. Hence, this paper seeks to delve into the changing trends in physical fitness among medical students during their university years and its correlation with their subsequent career paths.

## Methods

### Methodology and study design

This study employed a retrospective data analysis method, retrospectively selecting and analyzing data from the university's management database [20, 21]. The subjects of the study were undergraduate students from the West China School of Medicine (West China Hospital) Sichuan University [22, 23], spanning disciplines such as Clinical Medicine, Nursing, and Medical Technology. The sample consisted of 634 students, predominantly of Han ethnicity, aged between 20 and 32 years. They were enrolled in both 4-year and 8-year programs, the eight-year integrated program in clinical medicine in China offers a comprehensive medical education pathway, transitioning students from undergraduate medical studies through to the attainment of a Doctor of Medicine degree [24].

Chinese university students are mandated to complete four physical fitness tests during their university studies [25], typically conducted once or twice annually. The majority of students undergo their inaugural test in their freshman year and undertake the fourth one in their final year. A comparison of the medical students' physical fitness results from the first to the fourth test allows us to discern the trajectory of their health status changes. An analysis of the fitness data juxtaposed against the career paths in the employment database determines whether students' physical fitness influences their post-graduation outcomes.

### Data collection

The physical fitness tests are officially organized by the university, and all Chinese students are required to participate [25, 26]. The university strictly adheres to the physical fitness testing standards set by the Chinese government, ensuring a nationally unified testing procedure and measurement criteria, which guarantees the quality of the data. The test items for males and females are not identical, and the criteria for satisfactory results differ between genders. International students are exempted from the physical fitness tests, hence the data is solely from Chinese students. We collected the complete physical examination data for 634 undergraduates from the West China School of Medicine Sichuan University who graduated in 2022. The timeframe for these tests spanned from 2016 to 2022. The tests included: height, weight, lung capacity, 50-m sprint, sitting forward bend, standing long jump, sit-ups (for females), pull-ups (for males), 800-m run (for females), 1000-m run (for males), and BMI (which calculated by height and wight).

The employment system is designed by Chinese universities to record the career paths of all Chinese students. Every graduating student is required to input their employment or further study status into the system. Both the school and the national authorities rigorously review this data to ensure its accuracy. We collected the entire post-graduation outcome data for 634 undergraduates from the West China School of Medicine Sichuan University who graduated in 2022. This primarily falls into three main categories: pursuing further studies, employment, and undecided.

### Data analysis

The physical fitness data and employment data were matched using student ID numbers. An analytical database was established using Excel software, and the data integrity was checked. Data that did not meet the inclusion criteria, such as students who did not complete their studies due to dropping out, were excluded. Data analysis was conducted using SPSS (version 27.0) [27]. Quantitative data were described with means and standard deviations, and differences were analyzed using the t-test. Qualitative data were expressed in terms of numbers and percentages, and differences were assessed using the Chi-square test.

BMI was calculated using height and weight data. The post-graduation outcomes data were categorized into two groups. Students who found employment or pursued further studies at renowned hospitals, universities, or institutions were categorized as 'top'. Meanwhile, students who pursued opportunities at ordinary hospitals, universities, or institutions, as well as those who had not yet determined their post-graduation plans, were categorized as 'normal'.

## Results

### Demographics

In Table 1, we present the demographic characteristics of the participants. The study encompassed physical fitness data from 634 students. Of these, 249 (39.27%) were male, and 385 (60.73%) were female. Participants' ages spanned from 20 to 32 years, with a mean age of 23.06 ± 1.14 years, calculated as of 2022. A predominant majority, 537 (84.70%), identified as Han ethnicity. By academic program, 211 (33.28%) were enrolled in the 5-year Clinical Medicine track, 172 (27.13%) in the 8-year Clinical Medicine track, 177 (27.92%) in Medical Technology, and 74 (11.67%) in Nursing.

**Table 1:** Demographic characteristics

Variable	Number of Participants	Percentage (%)
**Gender**		
Male	249	39.27
Female	385	60.73
**Major**		
Nursing	74	11.67
Clinical Medicine (5 years)	211	33.28
Clinical Medicine (8 years)	172	27.13
Medical Technology	177	27.92
**Ethnicity**		
Han	537	84.70
Uighur	16	2.52
Tujia	12	1.89
Others	69	10.89
**Age**		
≤ 21	31	4.89
22–24	558	88.01
≥ 25	45	7.10

### Results of the four physical fitness tests

Table 2 illustrates the grade distribution of students during each of the four physical fitness tests. Notably:

- Six hundred eighteen students, accounting for 97.48%, underwent their first test in the freshman year.
- For the second test, a combined total of 555 students (87.54%) participated in either their freshman or sophomore years.
- The third test saw 425 students (67.04%) taking part in their sophomore or junior years.
- Finally, 442 students (69.72%) participated in the fourth test during their fourth or fifth years.

**Table 2:** Distribution of students by grade for each physical fitness test

Test sequence	Freshman	Sophomore	Junior	Senior	5th Year	6th Year
1st Test	618 (97.48%)	9 (1.42%)	1 (0.16%)	5 (0.79%)	1 (0.16%)	
2nd Test	408 (64.35%)	147 (23.19%)	37 (5.84%)	35 (5.52%)	7 (1.10%)	
3rd Test		177 (27.92%)	248 (39.12%)	142 (22.40%)	61 (9.62%)	6 (0.95%)
4th Test			177 (27.92%)	206 (32.49%)	236 (37.22%)	15 (2.37%)

**Fig. 1:**
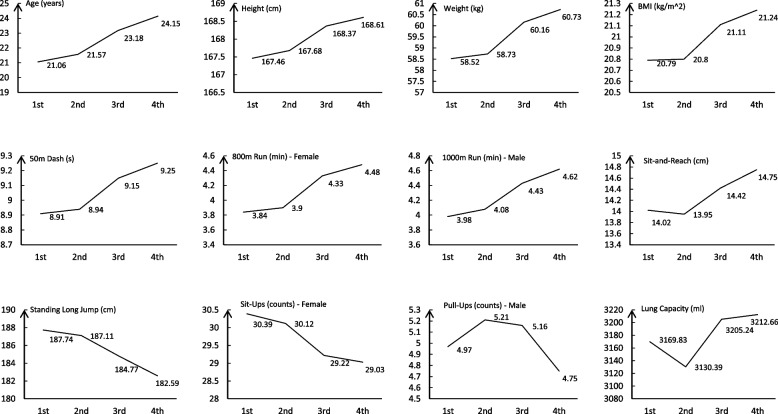
Trend of physical fitness changes in medical students

The comprehensive results of the quartile physical evaluations conducted on medical undergraduates are detailed in Table 3, with the longitudinal trend visualized in Fig. 1. A comparative analysis highlighted the stability of height measurements across all evaluations. A discernible distinction was observed in weight metrics: the initial evaluation registered an average weight of 58.52 ± 10.48 kg, escalating to 60.73 ± 12.07 kg by the fourth evaluation (*P* < 0.00). This weight augmentation led to a statistically significant variation in BMI, transitioning from 20.79 ± 2.74 kg/m^2 in the first assessment to 21.24 ± 3.06 kg/m^2 in the final one (*P* = 0.01). Pulmonary volume increased (3169.83 ± 819.39 ml to 3212.66 ± 831.33 ml, *P* = 0.03). Decline in agility and speed was evidenced in the running metrics: 50-m dash (from 8.9 ± 0.99 s to 9.25 ± 1.11 s, *P* < 0.00), 800-m run for females (from 3.84 ± 0.47 min to 4.48 ± 0.85 min, *P* < 0.00), and 1000-m run for males (from 3.98 ± 0.64 min to 4.62 ± 0.87 min, *P* < 0.00). The standing long jump exhibited a reduction in performance, marking a decrease from 187.74 ± 30.98 cm to 182.59 ± 32.26 cm (*P* = 0.02), and sit-ups for females decreased (30.39 ± 7.5 counts to 29.03 ± 8.82 counts, *P* < 0.00). Pull-ups for males remained statistically invariant. Sit-and-reach increased (14.02 ± 6.53 cm to 14.75 ± 6.65 cm, *P* < 0.00). Overall, the data underscores a trend of weight gain among undergraduates and a decline in muscular endurance, with flexibility remaining relatively constant.

**Table 3:** Results from the four physical fitness tests conducted on undergraduate medical students

Variables (Units)	1st Test	2nd Test	3rd Test	4th Test	*P*-value
Age (years)	21.06 ± 69.5	21.57 ± 69.48	24.15 ± 69.5	24.15 ± 69.5	0.00*
Height (cm)	167.46 ± 8.48	167.68 ± 8.53	168.37 ± 8.55	168.61 ± 8.58	0.00*
Weight (kg)	58.52 ± 10.48	58.73 ± 10.77	60.16 ± 11.66	60.73 ± 12.07	0.00*
BMI (kg/m^2)	20.79 ± 2.74	20.8 ± 2.82	21.11 ± 2.96	21.24 ± 3.06	0.00*
Lung Capacity (ml)	3169.83 ± 819.39	3130.39 ± 785.16	3205.24 ± 829.18	3212.66 ± 831.33	0.03*
Sit-and-Reach (cm)	14.02 ± 6.53	13.95 ± 6.35	14.42 ± 6.66	14.75 ± 6.65	0.00*
Standing Long Jump (cm)	187.74 ± 30.98	187.11 ± 30.78	184.77 ± 32.28	182.59 ± 32.25	0.00*
50m Dash (s)	8.91 ± 0.99	8.94 ± 1.02	9.15 ± 1.08	9.25 ± 1.11	0.00*
800m Run (min)—Female	3.84 ± 0.47	3.9 ± 0.57	4.33 ± 0.82	4.48 ± 0.85	0.00*
1000m Run (min)—Male	3.98 ± 0.63	4.08 ± 0.62	4.43 ± 0.97	4.62 ± 0.87	0.00*
Sit-Ups (counts)—Female	30.39 ± 7.5	30.12 ± 7.98	29.22 ± 8.83	29.03 ± 8.82	0.00*
Pull-Ups (counts)—Male	4.97 ± 4.63	5.21 ± 4.95	5.16 ± 5.69	4.75 ± 5.12	0.38

Differences between the 1st and 4th tests were analyzed using paired t-tests

### Post-graduation outcomes

To facilitate a comprehensive analysis, we have dichotomized the employment and further study data into two distinct categories: "top" and "normal" in Table 4. Institutions designated as "top" for further studies include those ranked within the QS World University Rankings' top 100, such as the University of Manchester, New York University, University of Queensland, and Brown University. Additionally, prominent Chinese institutions, namely Peking Union Medical College, Peking University, Fudan University, Shanghai Jiao Tong University, Sichuan University, and Sun Yat-sen University, also qualify under this category. Conversely, institutions ranked outside the QS top 100 or those with lesser recognition within China are classified as "normal". For employment outcomes, prominent Chinese medical institutions, including the West China Hospital of Sichuan University, Tongji Medical College Hospital of Huazhong University of Science & Technology, Xiangya Hospital of Central South University, and China-Japan Friendship Hospital, are categorized as "top". Positions like research assistants and roles within smaller enterprises and hospitals are designated as "normal".

**Table 4:** Career paths category

Career paths category	Further studies	Employment
Top	**QS World University Rankings' Top 100:** Includes institutions like the University of Manchester, New York University, University of Queensland, Brown University, etc **Prominent Chinese Institutions:** Includes Peking Union Medical College, Peking University, Fudan University, Shanghai Jiao Tong University, Sichuan University, Sun Yat-sen University, etc	**Prominent Chinese Medical Institutions:** Includes West China Hospital of Sichuan University, Tongji Medical College Hospital of Huazhong University of Science & Technology, Xiangya Hospital of Central South University, China-Japan Friendship Hospital, etc
Normal	Institutions ranked outside the QS top 100 or those with lesser recognition within China	Roles such as research assistants, positions in smaller enterprises and hospitals

**Fig. 2:**
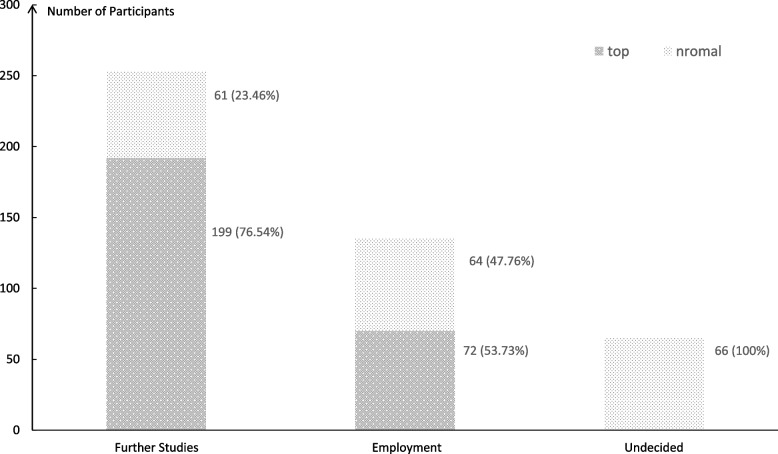
Count and proportion of individuals by different career paths

It's worth noting that the eight-year clinical medicine program sees students transition from undergraduate to postgraduate studies in their fifth year. Students from China Hong Kong, Macau, and Taiwan have significantly different career paths compared to those from mainland China. Therefore, the aforementioned students were excluded. The remaining 453 students were included in the analysis. We found that 253 students (55.85%) opted for further studies, 135 (29.80%) embarked on employment, while 65 (14.35%) remained undecided. The distribution of students between the "top" and "normal" categories for each outcome is depicted in Fig. 2.

### Association between physical fitness and career paths

Upon analyzing the physical assessment data from the first and fourth evaluations in Table 5, students on both "top" and "normal" career paths showed increased BMI and vital lung capacity, while their performance in running tests decreased.

**Table 5:** Physical fitness changes from 1st to 4th test for top and normal career paths

Variables (Units)	Career paths	1st Test	*P*-value	4th Test	*P*-value
**BMI (kg/m^2)**	top	20.96 ± 2.9	0.04*	21.48 ± 3.4	0.00*
	normal	20.46 ± 2.43		20.66 ± 2.51	
**Lung Capacity (ml)**	top	3141.05 ± 786.78	0.22	3248.31 ± 868.92	0.04*
	normal	3051.6 ± 772.52		3090.24 ± 737.2	
**50m Dash (s)**	top	8.92 ± 1.22	0.31	9.3 ± 1.11	0.42
	normal	9.02 ± 0.94		9.38 ± 1.01	
**Sit-and-Reach (cm)**	top	13.47 ± 6.5	0.03*	14.52 ± 6.31	0.39
	normal	14.79 ± 6.47		15.07 ± 7.01	
**Standing Long jump (cm)**	top	184.35 ± 31.08	0.84	181.3 ± 33.76	0.47
	normal	184.92 ± 29.05		179.19 ± 28.39	
**Pull-Ups (counts)—Male**	top	4.75 ± 4.54	0.90	5.05 ± 5.13	0.40
	normal	4.84 ± 4.76		4.37 ± 5.09	
**Sit-Ups (counts)—Female**	top	28.71 ± 7.51	0.00*	27.81 ± 9.41	0.07
	normal	31.27 ± 7.23		29.75 ± 8.87	
**1000m Run (min)—Male**	top	4 ± 0.81	0.86	4.7 ± 0.91	0.38
	normal	3.98 ± 0.63		4.59 ± 0.71	
**800m Run (min)—Female**	top	3.91 ± 0.45	0.20	4.44 ± 0.72	0.65
	normal	3.84 ± 0.52		4.48 ± 0.9	

Differences between the top and normal paths were analyzed using the independent samples t-test

To understand who experienced more significant changes, we calculated the difference by subtracting the first evaluation from the fourth. This transformed the continuous data into categorical outcomes ("Increase" or "Stable/Decrease"). A chi-square test comparing these changes to career paths revealed that while "top" students had slightly lesser changes in health metrics than "normal" students, only the change in BMI was statistically significant (*P* = 0.04). Notably, students with stable or reduced BMI during college had better post-graduation outcomes compared to those with increased BMI (47.30% to 37.70%, *P* = 0.04) in Table 6.

**Table 6:** Association of BMI change (4th to 1st) with career paths

BMI change (4th—1st)	Top	Normal	*P*-value
Increase	72(37.70%)	119(62.30%)	0.04*
Stable/Decrease	124(47.30%)	138(52.70%)	

Differences were analyzed using the chi-square test

## Discussion

In this study, we analyzed the physical fitness changes during university years among 634 undergraduate medical students and further examined the correlation between these physical fitness changes and their career paths. Physical fitness is linked to various aspects of a university student's performance, including academic studies and research. Elevating students' physical fitness levels has reached a consensus on an international scale. However, our findings indicate that the physical fitness of undergraduate medical students generally deteriorated during their university years. This decline was evident in factors such as weight gain, increased BMI, longer durations in both long-distance and short-distance runs, reduced standing long jump distances, diminished abdominal muscle endurance, and reduced upper body muscular endurance. The majority of students opted for postgraduate studies after completing their undergraduate degree. Students with a “normal” post-graduation path, compared to those heading to "top" paths, had better BMI, Sit-and-Reach, and Sit-Ups performances during their initial physical fitness test. However, by the time of the final test, only the BMI advantage remained for those heading to "normal" paths compared to their "top" peers. Comparing the differences between the results of the final and the initial physical fitness tests with career paths, we found that students whose BMI remained stable or decreased were more likely to head to "top" paths after graduation, and this difference was statistically significant.

In addition to physical fitness, the post-graduation paths of medical students are influenced by a variety of factors, such as demographic elements that were significant in our study, including gender, major, and ethnicity. However, regarding the changes in physical fitness during university years, the only fitness parameter in our study that showed a statistically significant correlation with post-graduation paths was BMI.

The post-graduation path is the culmination of university studies, and achieving excellent academic performance is often a key to success. Research indicates that physical exercise not only enhances physical health but also improves academic performance [28]. Students with better physical fitness tend to have higher academic achievements [29], with cardiorespiratory fitness being positively associated with academic success [30]. This is supported by cohort studies from the Uniformed Services University in the United States, which also show positive correlations between physical fitness and success in medical studies [8].

### Medical students' physical fitness requires increased attention

While the physical fitness of university students globally has been a subject of broad concern, with countries implementing various measures to enhance their students' fitness levels, our research suggests a noticeable decline in the fitness status of medical students during their university years. Another studies in China and US also find the overall physical fitness of college students is on a downward trend [31, 32].. Across all tested metrics (with the exceptions of lung capacity and sit-and-reach), there was a decline. Notably, there was a significant increase in weight, while muscle endurance, running, and jumping capabilities all saw a notable decrease.

Weight plays a crucial role in affecting student's athletic abilities [33]. Performance in events linked with muscle endurance, such as sprints, long-distance running, long jump, sit-ups, and pull-ups, are influenced by weight. On the other hand, the sit-and-reach, which measures body flexibility, has a correlation with height and is less affected by weight. Lung capacity, related to chest cavity development, is also less influenced by weight. Therefore, the scores for lung capacity and sit-and-reach d both increased for medical students during their university tenure.

The decline in physical fitness among medical students during their university years could be attributed to dietary changes leading to weight gain, coupled with decreased physical activity, leading to diminished athletic abilities. The shift in dietary habits may arise from students transitioning from family life to campus life, with fewer familial constraints and freer lifestyle [34]. The decline in physical activity might be due to the intense academic pressures faced by medical students, devoting more time to their studies and neglecting exercise and sports [35]. Moreover, a lack of physical activity can further increase the risk of obesity [36].

### Students headed for top career paths demonstrate better physical fitness changes than those heading for normal paths

At the initial stage, students destined for normal paths had a distinct advantage in physical fitness over those aiming for top paths. However, this advantage was not maintained until graduation. Looking retrospectively, during the first fitness test, students heading for normal paths outperformed those headed for top ones in terms of BMI, Sit-and-Reach, Standing Long Jump, Pull-Ups – Male, Sit-Ups – Female, 1000 m Run – Male, and 800 m Run – Female. Specifically, statistically significant differences were found in BMI, Sit-and-Reach, and Sit-Ups – Female. However, by the final fitness test, the advantages were only seen in BMI, Sit-and-Reach, Sit-Ups- Female, and 1000 m Run – Male, with only BMI showing a statistically significant difference. Conversely, by the last test, students aiming for top paths displayed superior results in Lung Capacity, 50 m Dash, Standing Long Jump, Pull-Ups – Male, and 800 m Run – Female when compared to their peers aiming for normal paths, with only Lung Capacity showing statistical significance. This indicates that students heading for top career paths maintained better physical fitness throughout their university years compared to those headed for normal paths. This could be linked to the former's superior study and lifestyle habits. These students set higher standards for themselves, evident not only in academics but also in their commitment to exercise, whereas those headed for normal paths may have become more complacent in their physical conditioning during their university years.

### Good physical fitness among university students can pave the way for a brighter future

Our research indicates that students whose BMI change (from the 1st to the 4th test) remains stable or declines are more likely to secure top career paths compared to those who experienced an increase in BMI change (47.3% to 37.7%, *P* = 0.04). Given that BMI is calculated based on weight and height, and that the height of medical students generally increased during their university years, a rise in BMI can be attributed to weight gain. This suggests that weight gain can influence the post-graduation prospects of medical students. Those who manage to maintain or slightly reduce their weight during their academic tenure tend to be more competitive upon graduation. Aside from BMI, other physical fitness test metrics did not show statistically significant differences in their influence on post-graduation outcomes for medical students.

### Universities should do more to improve the physical fitness of medical students

Universities should take more initiatives to enhance the physical fitness of medical students. This is not only related to their health and academic performance but also has implications for their career development after graduation. Some schools have already begun taking action. For instance, the West China School of Medicine at Sichuan University, starting from 2021, has mandated that all first and second-year students complete a 3-km run five times each semester to boost their physical condition. With encouragement and requirements from the institution, students are more likely to actively participate in physical exercises.

## Conclusion

Through analyzing the changes in the physical fitness of medical students during their university years, our study has uncovered the decline in the physical health of medical students. We also explored the correlation between changes in physical health and career paths, highlighting that variations in BMI can influence their post-graduation outcomes. This research contributes to a comprehensive understanding of the physical fitness status and changing trends of medical students and its impact on their career development. We emphasize the importance of physical fitness exercises for university students and call upon universities to take further measures to enhance the physical fitness of medical students.

## Limitations of study

The health awareness of medical students might impact their post-graduation paths. However, as this study is based on database analysis, it lacks survey data on the students' psychology and therefore cannot be analyzed in this context. Additionally, the data in this study are solely from Sichuan University, which limits the generalizability of the conclusions.

## Data Availability

The datasets used and analyzed in this study are available upon reasonable request. The corresponding author can be contacted for access.
